# Optimization of the Thermal Performance of Na_2_HPO_4_·12H_2_O-Based Gel Phase Change Materials in Solar Greenhouses Using Machine Learning

**DOI:** 10.3390/gels11090744

**Published:** 2025-09-16

**Authors:** Wenhe Liu, Xuhui Wu, Mengmeng Yang, Yuhan Huang, Zhanyang Xu, Mingze Yao, Yikui Bai, Feng Zhang

**Affiliations:** 1 College of Ocean and Civil Engineering, Dalian Ocean University, Dalian 116023, China; liuwenhe@dlou.edu.cn; 2 College of Engineering, Shenyang Agricultural University, Shenyang 110866, China; 3 College of Water Conservancy, Shenyang Agricultural University, Shenyang 110866, China; 2024240175@stu.syau.edu.cn (X.W.); 2022166045@stu.syau.edu.cn (Y.H.); xuzhanyang@syau.edu.cn (Z.X.); yaomingze@syau.edu.cn (M.Y.); baiyikui@syau.edu.cn (Y.B.); fzhang2019@syau.edu.cn (F.Z.)

**Keywords:** gel phase change material, support vector regression, random forest, gradient boosting trees, thermal properties optimization

## Abstract

In the design of gel phase change composite wall materials for solar greenhouses, the alteration of material composition could directly affect the thermal performance of gel phase change composite wall materials. In order to obtain better suitable gel phasechange composite wall material for solar greenhouses, Na_2_HPO_4_·12H_2_O-based gel phasechange materials with different content of ingredient (Na_2_SiO_3_·9H_2_O, C_35_H_49_O_29_, KCl, and nano-α-Fe_2_O_3_) were obtained via the Taguchi method and machine learning algorithms, such as Support Vector Regression (SVR), Random Forest (RF), and Gradient Boosting Trees (GBDT). The result shows that the GBDT is more suitable for the thermal performance optimization prediction of gel phase change composite wall materials, including time cooling (TC), latent heat of phase change (ΔH_m_), supercooling degree (ΔT), and phase change temperature (T_m_). The determination coefficient (R^2^) of time cooling (TC), latent heat of phase change (ΔH_m_), supercooling degree (ΔT), and phase change temperature (T_m_) by GBDT are 0.9987, 0.99965, 1, and 0.9995, respectively. The mean absolute error (MAE) coefficient percentage of supercooling degree (ΔT), phase change temperature (T_m_), latent heat of phase change (ΔH_m_), and time of cooling (TC) by GBDT are 0.32%, 0.25%, 0.17%, and 0.26%, respectively. The root mean square error (RMSE) of supercooling degree (ΔT), phase change temperature (T_m_), latent heat of phase change (ΔH_m_), and time of cooling (TC) by GBDT are 0.41%, 0.32%, 0.19%, and 0.35%, respectively. The optimal result predicted by GBDT is Na_2_HPO_4_·12H_2_O + 5% Na_2_SiO_3_·9H_2_O + 12% KCl + 0.2% Nano-α-Fe_2_O_3_ + 3% C_35_H_49_O_29_, which was verified by experiments.

## 1. Introduction

The Chinese solar greenhouse (CSG) was a unique type of horticultural facility in Northern China, characterized by high efficiency, energy savings, and low cost [1]. The CSG features a building envelope mainly composed of three components: the south roof, the north roof, and the north wall [2,3]. This special structural design is beneficial for maximizing solar energy utilization while minimizing heat loss. The north wall stores surplus thermal energy during daylight hours and subsequently releases it back into the greenhouse at night, thereby maintaining nocturnal temperatures [4] and reducing or even eliminating the supplemental heating needs of the CSG in winter. The north wall was crucial for enhancing solar energy utilization and reducing nighttime heat loss, which ultimately decreased heating needs and improved thermal environment [2]. However, the overall thermal storage capacity of greenhouses in Northern China was constrained by several inherent factors, primarily including ambient temperature, the intensity and duration of solar radiation, as well as the thermal storage performance of the walls [5]. Despite elevated indoor temperatures during the day, the stored thermal energy often experienced significant depletion after midnight—especially during harsh winter periods—failing to meet the thermal requirements for crop development [6].

Various measures were proposed to regulate the microclimate of greenhouses in order to conserve energy, protect the environment, and reduce carbon emissions. Enhancing the thermal storage capacity and thermal resistance performance of the north wall was recognized as an effective approach to improving the thermal environment of CSG [7,8]. Ren et al. [9] developed a novel straw-based composite block (ST), composed of wheat straw fiber, sand, and cement, specifically designed for application on the north wall of solar greenhouses. Compared with traditional porous clay blocks (CL) of the same thickness, the ST block demonstrated enhanced thermal storage capacity and higher total thermal resistance, resulting in a 0.5 °C increase in indoor temperature during nighttime. In addition, Zhang et al. [9,10] employed five distinct materials in the construction of the northern wall, specifically gravel, gravel silt, concrete, concrete hollow blocks, and aerated concrete blocks. Experimental results indicated that the thermal storage and release performance of gravel–silt soil, concrete, and gravel walls improved by 5.0%, 38.0%, and 37.3%, respectively, compared to traditional rammed earth walls. The average indoor nighttime temperatures increased by 0.7 °C and 2.5 °C, respectively. The partial modification of the northern wall material could be carried out under relatively low-cost conditions, providing benefits in terms of construction efficiency and minimal maintenance requirements. Zhang et al. [11] investigated a multi-layered greenhouse wall structure comprising a 60 mm reinforced concrete slab, a 440 mm rammed earth layer, a 100 mm lightweight thermal insulation panel, and a 100 mm expanded polystyrene board. In this configuration, the rammed earth layer played a predominant role in thermal storage, followed by the inner reinforced concrete slab. Although the outer lightweight insulation board had insufficient thermal storage capacity, it was crucial for providing insulation. The incorporation of 100 mm expanded polystyrene boards significantly enhanced the wall’s thermal storage capacity by 25.0% and improved the heat release capacity to the concrete hollow blocks by 41.7%. This structural configuration considered both thermal storage and insulation performance, thereby achieving optimal efficiency. Liu et al. [12] introduced an innovative greenhouse structure, wherein the northern wall is insulated using expanded polystyrene cavity mold blocks, and the southern roof employs a water circulation system for thermal insulation. During a continuous period of seven days characterized by cold and overcast weather, the average nighttime minimum temperature in the newly constructed greenhouse was 10.1 °C, with a remarkable temperature difference of up to 38.0 °C between the interior and exterior of the winter greenhouse simulator in this greenhouse, which was further optimized to improve the heat storage and release of the system [13]. Similarly, Xia et al. [14] implemented magnetron sputtering collector plates within a water-circulation-based solar thermal system to supply heating for greenhouse environments. The sole energy consumption associated with the system was the electrical power required to drive the water pump, resulting in an average daily heat collection efficiency of 70.2%. This system demonstrated the capability to elevate indoor air temperature by an average of 3.5 °C during nighttime hours while maintaining temperatures above 9.0 °C on a continuous basis. In summary, the substantial thermal capacity of the northern wall played a crucial role in nocturnal heating within solar-powered greenhouse systems. The sensible heat storage system exhibited multiple advantages, including economic viability, considerable thermal storage capacity, broad environmental adaptability, and well-developed technical feasibility. However, the heat storage equipment was large in volume and experienced certain temperature fluctuations.

To enhance the thermal storage capacity of greenhouse walls, utilizing phase change materials (PCMs) was proposed as a construction material for the building envelope, aiming to improve energy efficiency. Previous research primarily focused on the application of organic phase change materials (PCMs) in the field of solar greenhouses [15,16,17,18,19]. However, organic PCMs exhibited drawbacks such as low phase change enthalpy [8], high material costs [20], and poor thermal conductivity [21], which significantly hindered applications in thermal management materials for CSG. In contrast, inorganic PCMs have advantages such as high thermal storage performance, fast thermal conductivity, non-flammability, being environmentally friendly and non-toxic, as well as lower costs [22,23,24,25,26]. These deficiencies were closely related to the preparation process of phase change materials [18]. However, inorganic salt materials exhibited several limitations, such as pronounced supercooling tendencies, high susceptibility to phase separation [27], and mismatches between phase transition temperatures and actual application requirements. These limitations were closely associated with the fabrication processes of phase change materials.

The incorporation of nucleating agents promoted the crystallization process, thereby effectively reducing the extent of supercooling. In hydrated salt phase change materials, thermostats were often added to weaken the interaction between salt and water molecules in hydrated salts to lower the melting point [28]. To mitigate the phase separation phenomenon, the integration of thickening agents has been proven to be an effective strategy [29,30]. Nanoparticles, which possessed nanoscale size effects, a large specific surface area, and strong interfacial interactions, had been incorporated into phase change materials (PCMs) to improve their thermal performance [31]. Tang et al. [32] utilized Na_2_SiO_3_·9H_2_O as a nucleating agent to suppress supercooling in PCMs and conducted an in-depth investigation into the mechanism through which nucleating agents affected the supercooling behavior of such materials. Fu et al. [33] incorporated KCl into the phase change system to regulate its phase transition temperature. The experimental results revealed that the phase transition temperature of the modified composite was markedly lower than that of the original material, showing a consistent decline with increasing KCl mass fraction. Lan et al. [34] proposed a novel gelation strategy for grafting (C_6_H_7_NaO_6_)_n_ onto (C_3_H_3_NaO_2_)_n_ within the Na_2_HPO_4_·12H_2_O molten salt system and demonstrated that this method could effectively inhibit phase separation through short-term thermal cycling. Wang et al. [35] investigated the influence of nano α-Fe_2_O_3_ incorporation on the thermal conductivity performance of phase change materials. The results showed that nano-α-Fe_2_O_3_ could effectively increase the thermal conductivity of Na_2_HPO_4_·12H_2_O, as illustrated by the fact that the addition of 0.2% nano-α-Fe_2_O_3_ increased the thermal conductivity by 90.8%. Yu et al. [36] conducted systematic research and development on Compressed Air Energy Storage (CAES) technology using orthogonal experimental design and the Technique for Order Preference by Similarity to Ideal Solution (TOPSIS). Meanwhile, Luo et al. [37] improved the supercooling and phase separation performance of hydrate phase change salts by incorporating varying mass fractions of sodium carboxymethyl cellulose (CMC) and xanthan gum (XG). Liu et al. [38] utilized Na_2_HPO_4_·12H_2_O as a baseline material, incorporating varying mass fractions of modified materials. A detailed analysis of the effects of these modified materials on the performance characteristics of the multiphase PCMs was conducted using the Taguchi design method, leveraging S/N ratios and variance analysis to identify the optimal formulation. However, traditional methods for the preparation of phase change materials often necessitated numerous repetitive experiments to identify high-performance materials. This approach was not only time-consuming but also required substantial investment in terms of human resources, materials, and costs.

Recently, the rise of science and technology, particularly in the field of machine learning, has brought significant changes to our daily lives and has become a powerful tool available to researchers. With the emergence of machine learning, the study of large datasets has become faster, easier, and clearer than before. Machine learning algorithms were already introduced for predicting the performance of phase change materials. For example, Mehrotra et al. [39] utilized the random forest method to study the phase change process of biopolymers (L-alanine and L-cysteine). By combining vibrational frequency data with breakpoint analysis, successful predictions of phase change temperature were achieved, which validated the effectiveness of the algorithm in phase change detection and demonstrated a significant improvement in the accuracy of phase change detection for biopolymers. Alade et al. [40] employed the Bayesian optimization method for support vector regression (BSVR) to fine-tune the hyperparameters of support vector regression using Bayesian algorithms. A model was trained with 84 sets of experimental data and validated with 17 sets of test data, successfully achieving the modeling and prediction of the specific heat capacity of aluminum oxide/ethylene glycol nanofluid. The results indicated that the BSVR model achieved a prediction accuracy of up to 99.95%, which significantly outperformed existing analytical models. G. Anooj et al. [41] trained a Gated Recurrent Unit (GRU) model using a dataset of 345 simulation samples to predict the liquid phase fraction of phase change materials under varying thermal inputs. The results indicated that the computational efficiency of the proposed approach surpassed that of traditional methods [41]. Ziapour et al. and colleagues used a Genetic Algorithm (GA) to optimize the application of Phase Change Materials (PCM) in solar greenhouse collectors [42]. Through simulations involving varying numbers of collectors and PCM volumes, they concluded that the optimized system significantly enhanced energy storage efficiency, reduced costs associated with diesel and natural gas, and improved overall energy utilization efficiency.

Traditional machine learning models often faced significant challenges when dealing with nonlinear problems or tasks that required large amounts of high-quality training. In emerging domains, such as renewable energy, the collection of extensive and high-quality training datasets was frequently constrained by privacy protection issues, strict regulatory frameworks, and the intrinsic complexity of the data. The collection of such fine-grained data involved significant privacy concerns, especially in cases requiring both traceable (personally identifiable) and untraceable (non-directly identifiable) measurements. Due to privacy concerns, users may be reluctant to provide detailed electricity consumption data, which could hinder the processes of data collection and analysis. Hameed et al. [43] indicated that there was a trend of wind farms transitioning from onshore to offshore development due to social and political factors. However, offshore wind farms faced several challenges in data collection, including high costs and data inconsistencies. Engaging professionals with profound expertise in system operations for data collection was imperative, as it not only ensures the accessibility and usability of data but also contributes to the safe and stable operation of offshore wind farms. Moreover, the presence of outliers in the database could lead to unreliable information. Therefore, addressing these outliers and accurately labeling the collected data was crucial for ensuring the effective operation of the data collection mechanisms, often requiring specialized knowledge in the specific field [44]. These challenges constrained our capacity to fully harness the potential of big data, thereby adversely impacting the training efficacy and performance of machine learning models. In light of these recognized challenges, the emerging research direction of small data learning has garnered increasing attention. This learning paradigm aimed to construct robust and reliable machine learning models under conditions of data scarcity or limitation [45,46].

The current paper employed three machine learning models (SVR, RF, GBDT) to predict the performance metrics of 54 additional modified gel PCMs, drawing on the collection and analysis of data from 27 modified material compositions and their associated performance indicators derived from a Taguchi experimental design featuring four factors at three levels L_9_(3^4^). The primary objective of this research was to validate the applicability of kernel methods or ensemble learning algorithms in supervised learning contexts, particularly for small sample sizes, nonlinear relationships, and multi-output data scenarios. Furthermore, this investigation sought to identify the model that achieved the highest fit with minimal error, thereby providing novel theoretical foundations and practical insights for optimizing the compositions and performance indicators of composite phase change materials. Finally, the gel phase change material with the most outstanding thermal performance for solar greenhouses was obtained.

## 2. Results and Discussion

### 2.1. Thermal Characteristics of PCMS Under Taguchi Experimental Design

**Figure 1:**
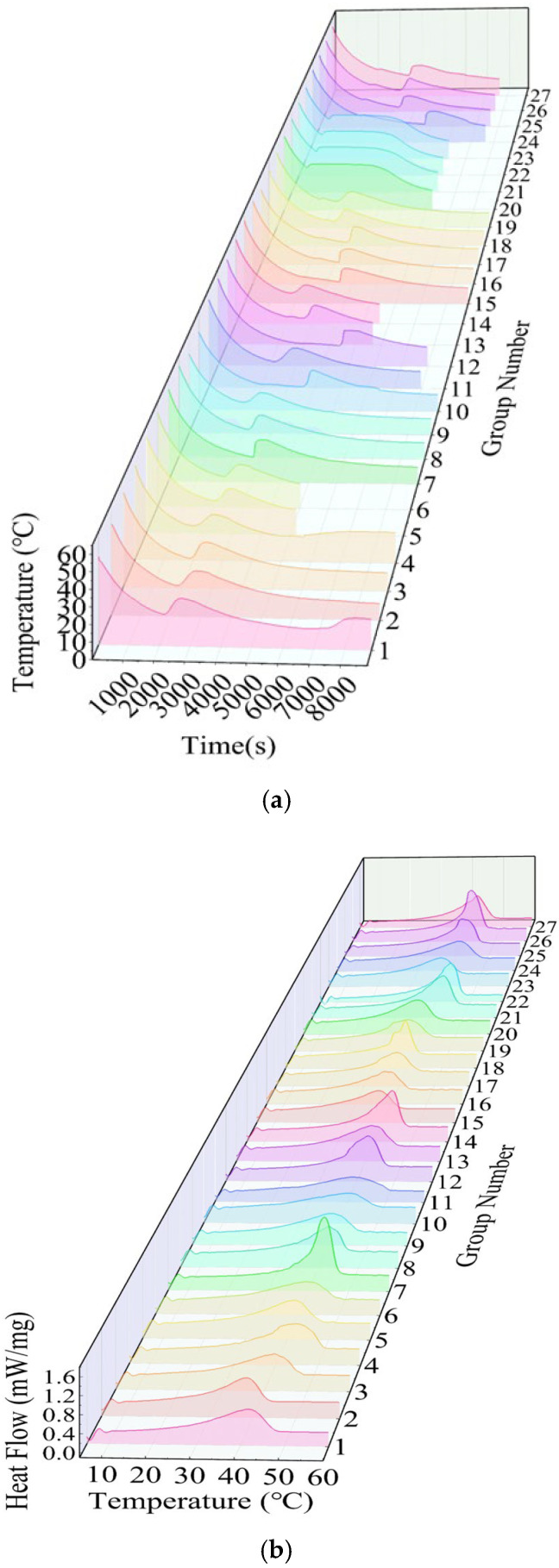
Experimental data visualization. (**a**) Twenty-seven sets of cooling curve charts. (**b**) Twenty-seven sets of DSC curves.

**Figure 2:**
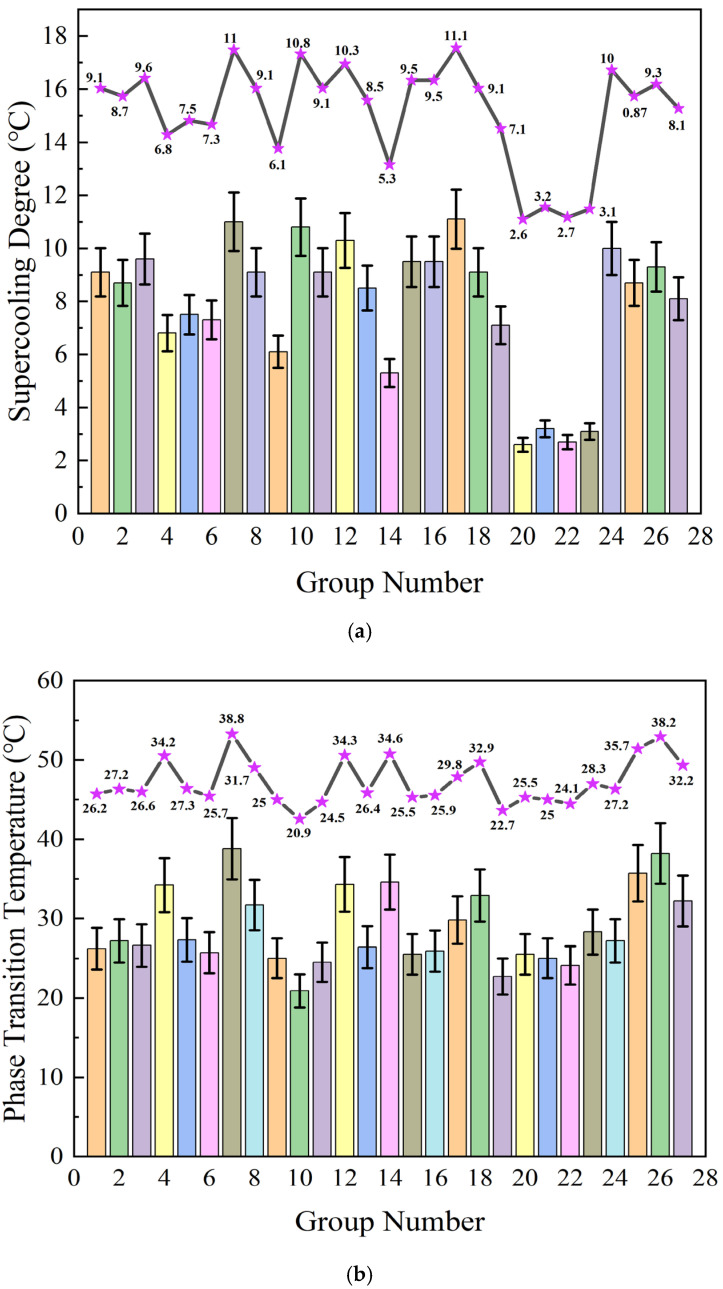

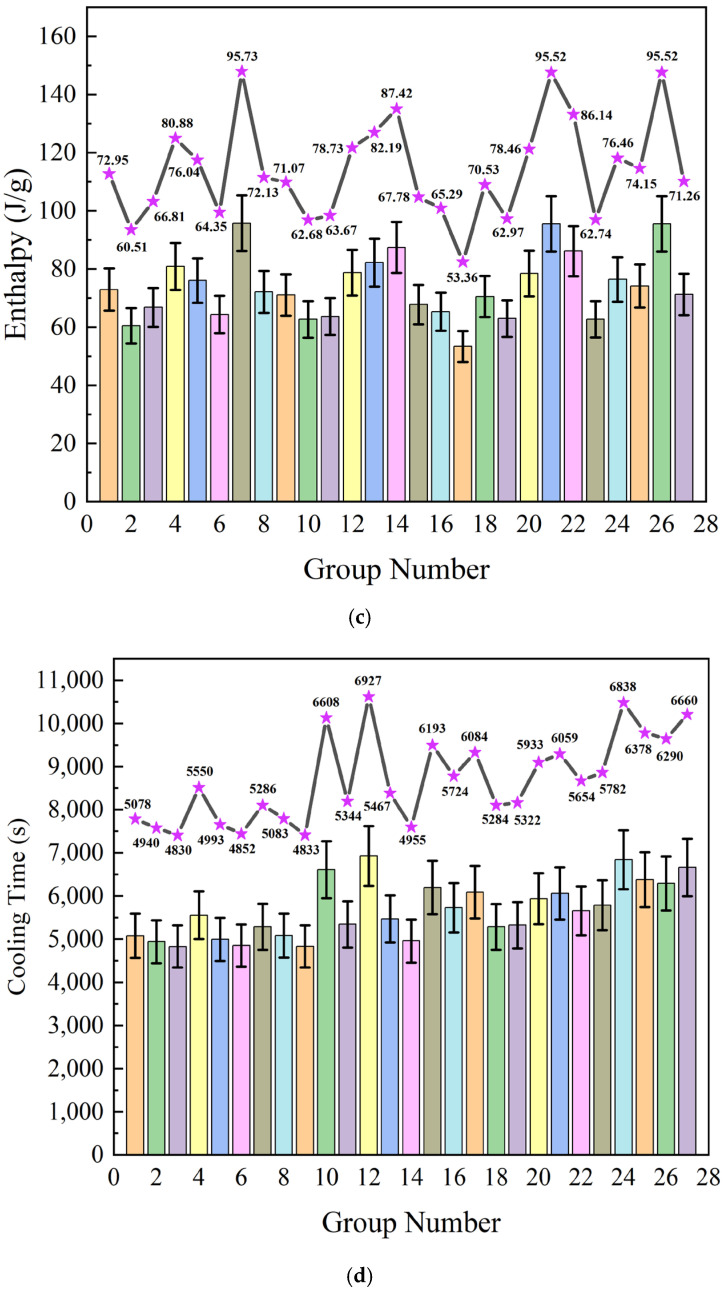
Performance Indicator Chart. (**a**) Supercooling degree; (**b**) phase change temperature; (**c**) phase change latent heat; (**d**) thermal conductivity rate. To present the column chart data more intuitively, a purple dotted line chart with stars was drawn above the column chart.

In the process of this experiment, the sample is placed in a sample vial, and the temperature probe is inserted into it without touching the bottom or walls of the vial. After heating and melting in a 60 °C constant-temperature water bath, the sample vial is placed into a low-temperature constant-temperature chamber pre-set to 10 °C. Every temperature change is recorded every 10 s using a multi-channel data logger to plot the cooling curve, as shown in Figure 1. The cooling rate is calculated based on the time required for the temperature to drop from 50 °C to 15 °C, and the degree of supercooling is calculated from the temperature change curve over time. The thermal performance of PCMs was measured using a differential scanning calorimeter (Netsch 214, DSC) from Shanghai, China. The scanning temperature range was 10–60 °C, with a scanning rate of 5 °C/min. DSC curves were plotted based on the measured data, and each data set was processed to obtain the corresponding phase change temperature and phase change enthalpy, as shown in Figure 2. Through a series of tests and data processing, the experimental results for supercooling degree (△T), phase change temperature (Tm), phase change latent heat (△Hm), and thermal conductivity rate (TC) are shown in Figure 2 and Table 1.

After a series of experimental treatments, this study selected the 20th experimental ratio based on the following criteria: a lower degree of supercooling, a shorter cooling time from 50 to 15 °C, a phase transition temperature within the range of 25 to 28 °C, and a higher latent heat of phase transition are preferable. The base material was Na_2_HPO_4_·12H_2_O, with the addition of 5 wt% SMN, 12 wt% KCl, 0.2 wt% nano-α-Fe_2_O_3_, and 3 wt% XG.

### 2.2. Optimization of PCMs Components by Machine Learning

#### 2.2.1. Factor Correlation Analysis

**Figure 3:**
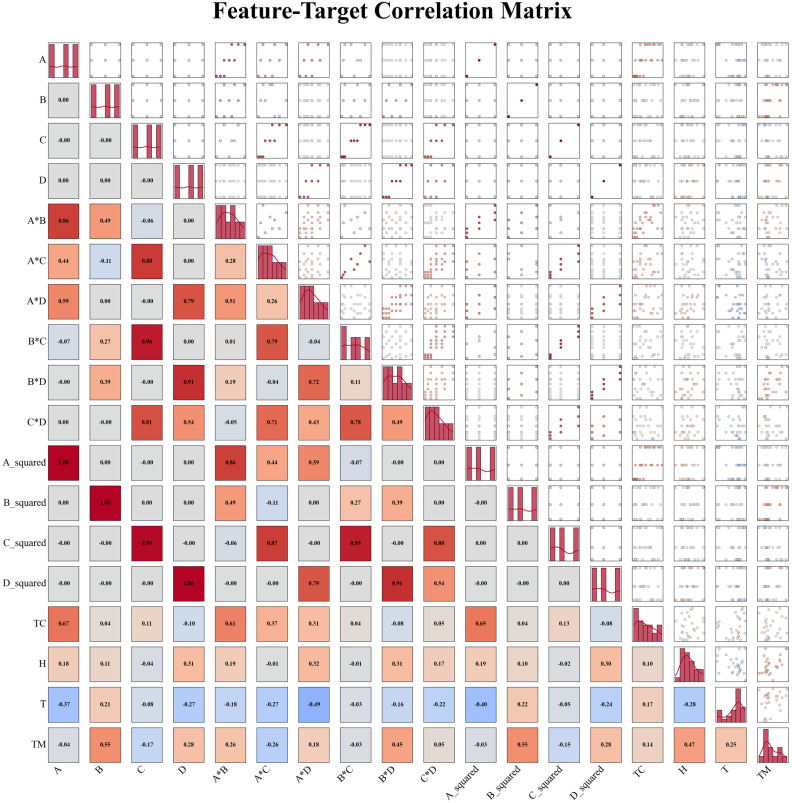
Feature–Target Correlation Matrix.

The heatmap quantifies relevant dimensions, providing intuitive and rigorous support for the analysis of experimental data. Not only can it reveal the relationship between factors and target values, but it can also guide model optimization, parameter selection, and multi-objective decision-making, making it a core tool in experimental design, data analysis, and engineering optimization. In machine learning, heatmaps assist in data preprocessing and feature engineering while also providing insights into the decision logic of models [40]. The Pearson correlation coefficient matrix and visualized factor–target correlation heatmap were calculated, as shown in Figure 3, which intuitively presents the correlation strength and direction between the four factors (A, B, C, D) and performance indicators (TC, H, T, TM). The heatmap represents the absolute value of the correlation coefficients with the depth of color (range [−1, 1]). Positive values indicate a positive correlation between factors and targets (as the factor level increases, the target value tends to increase), while negative values indicate a negative correlation [40]. By selecting factor–target pairs with high absolute correlation coefficients (e.g., |r| > 0.5), key factors that significantly influence the target can be quickly identified (such as the strong negative correlation between factor C and the T indicator).

#### 2.2.2. Machine Learning Analysis

**Figure 4:**
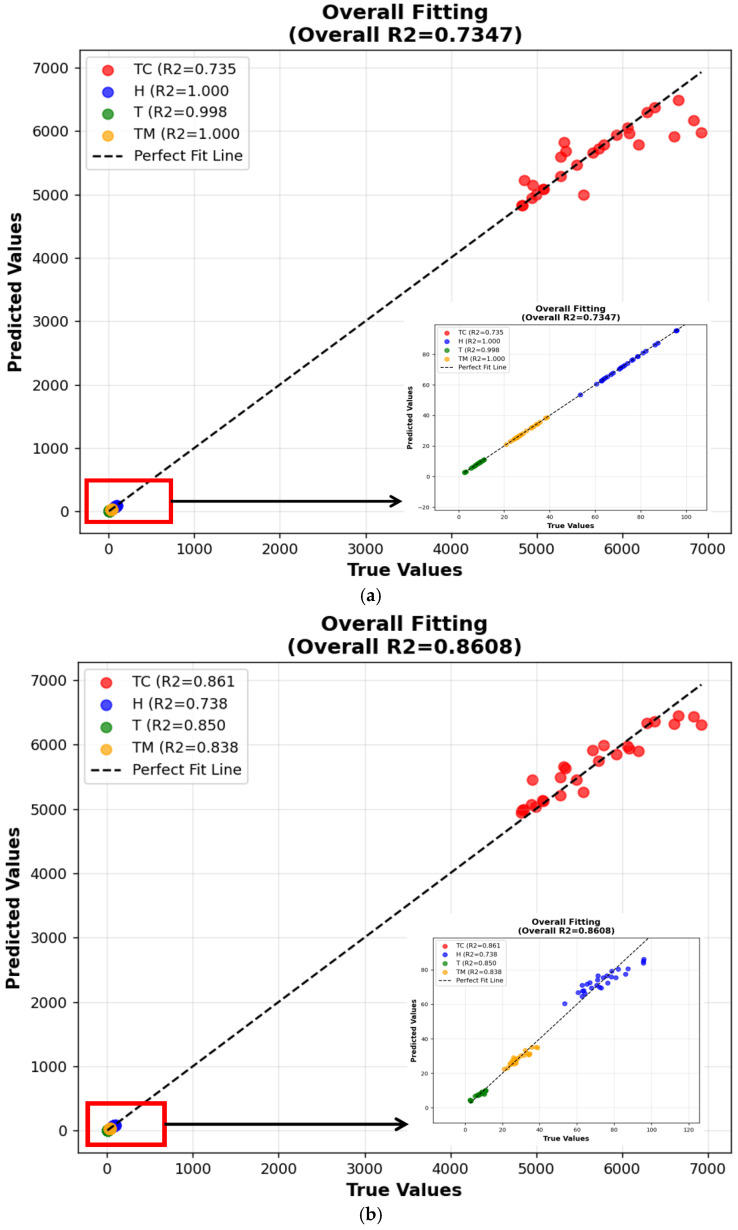

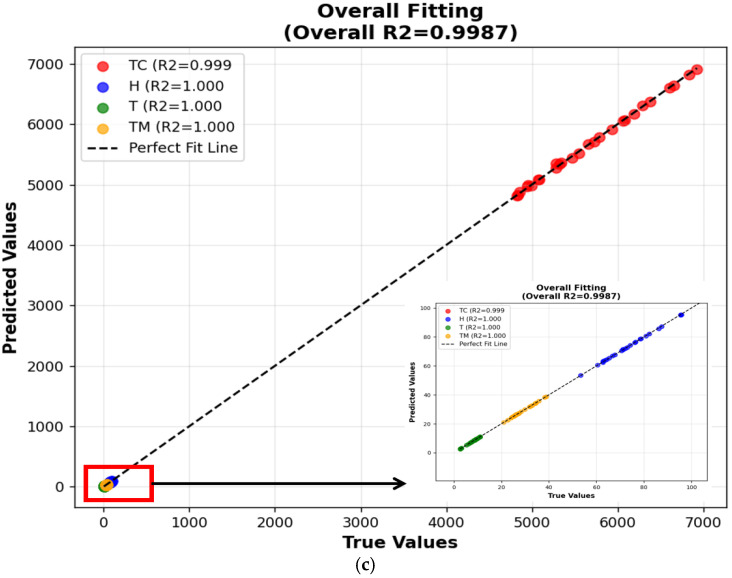
Overall fit score. (**a**) Support Vector Regression; (**b**) Random Forest; (**c**) Gradient Boosting Tree.

**Figure 5:**
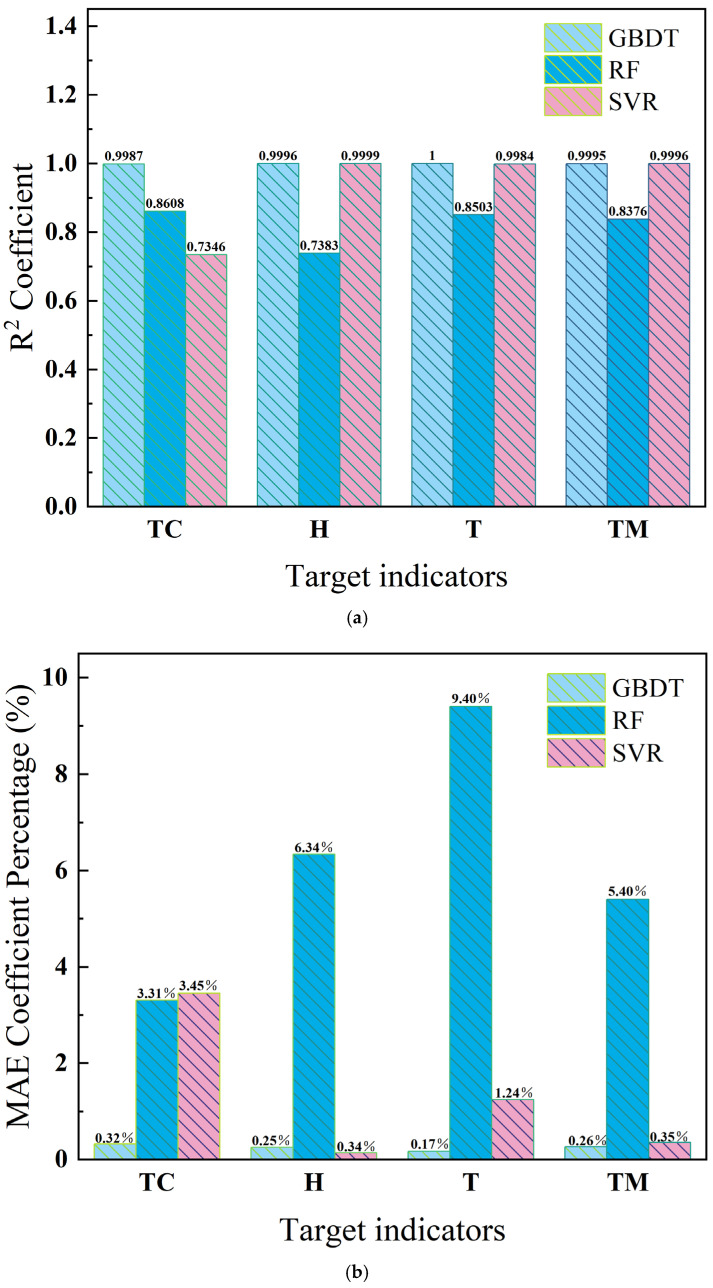

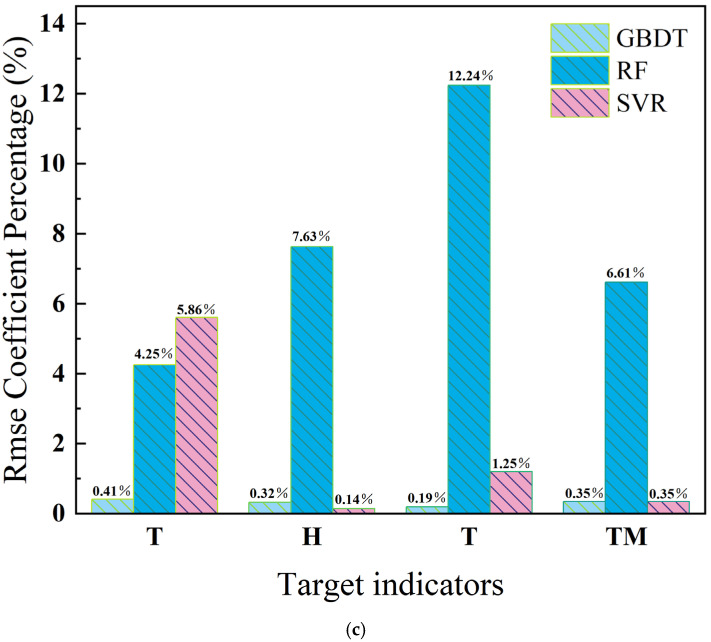
Evaluation indicators. (**a**) R^2^ Coefficient; (**b**) MAE Coefficient Percentage; (**c**) RMSE Coefficient Percentage.

To comprehensively evaluate the predictive performance of three machine learning algorithms in PCMs components, cross-validation and introduction of three evaluation metrics are conducted for a comprehensive score in model assessment, such as Mean Absolute Error (MAE), Root Mean Square Error (RMSE), and Coefficient of Determination (R^2^). The decision coefficient R^2^ is introduced to evaluate the scores of various indicators and verify the fitting effect of the model. In the engineering field, when the decision coefficient R^2^ is greater than 0.6, it indicates that the fitting effect is valid [41]. The closer the decision coefficient is to 1, the better the fitting effect and the higher the accuracy. Figure 4 and Figure 5 shows the R^2^ fitting graphs of three models, where SVR is 0.7347; RF is 0.8608, and GBDT is 0.9987. Therefore, the calculation effect of GBDT is the best.

### 2.3. Optimization Model Verification and New PCM Proposed

**Figure 6:**
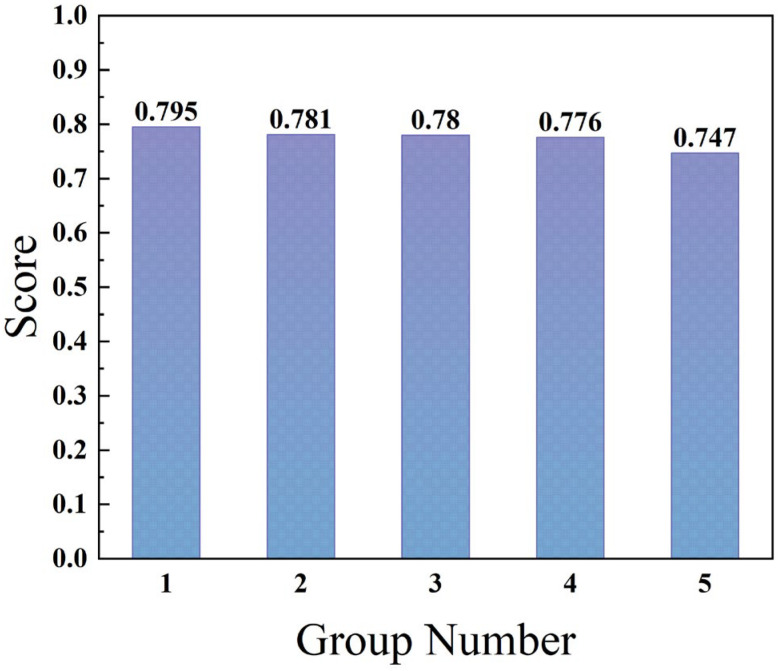
Comparison chart of overall scores.

**Figure 7:**
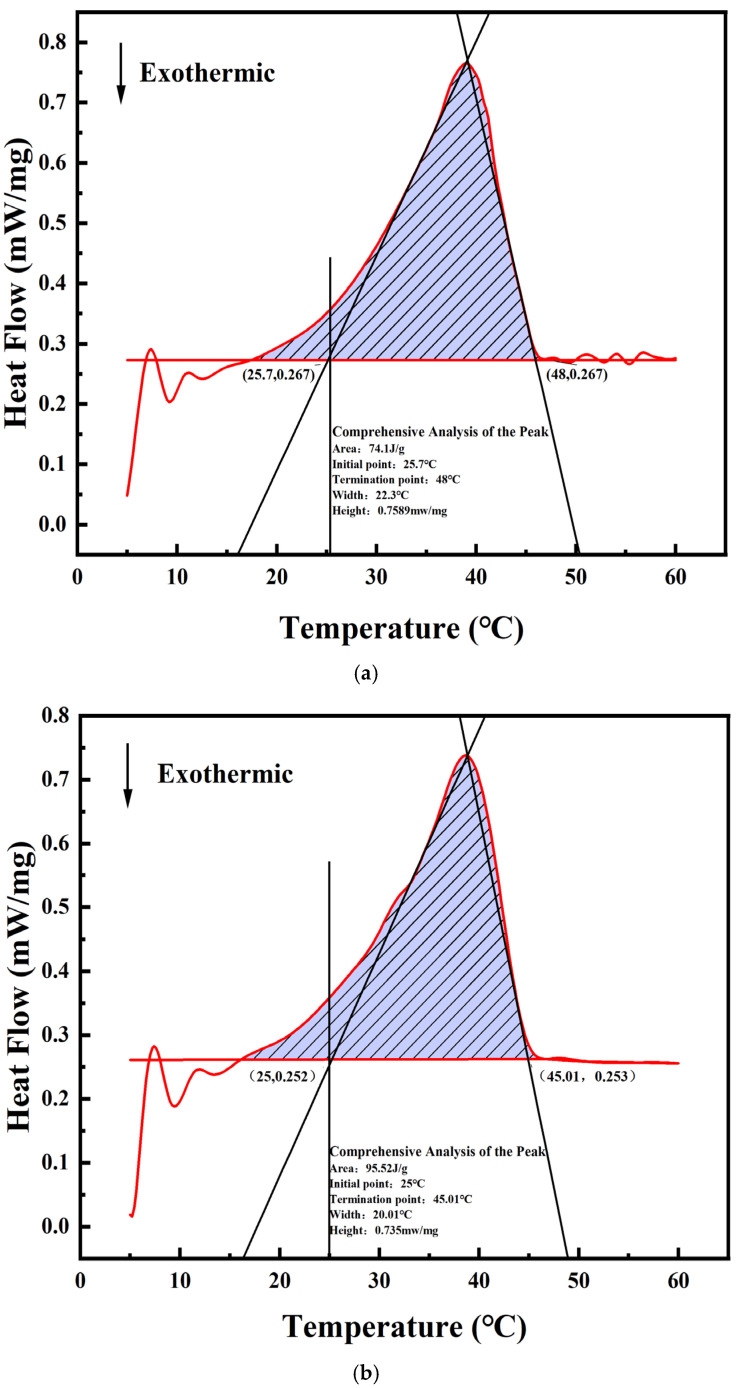

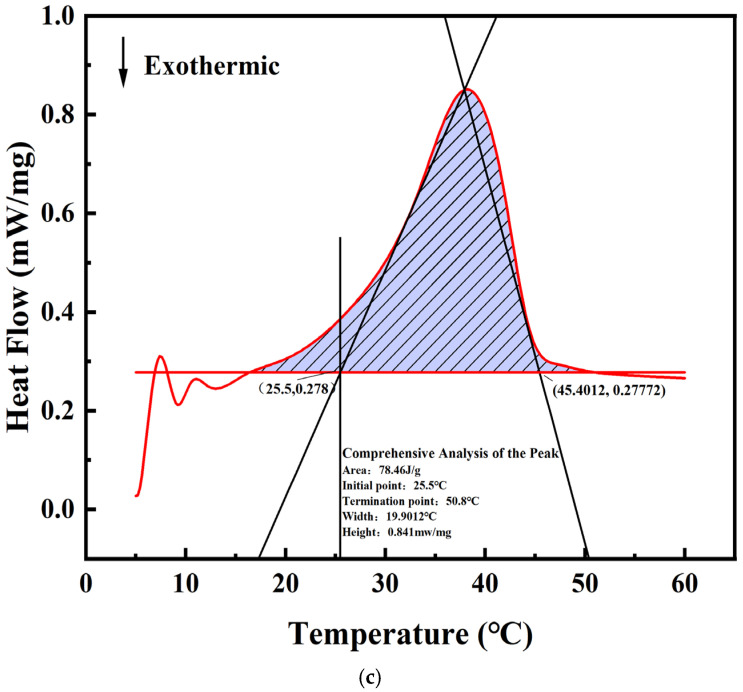
Three sets of optimal DSC curve graphs. (**a**) Forecast (Score 3); (**b**) Measured (Score 2); (**c**) Measured (Score 1).

To further verify the actual predictive ability of the model, the best-performing GBDT model is selected to predict the PCM data for further study. After data preparation and model training, the GBDT model is identified and predicted by 54 combinations. A multi-index comprehensive optimization method is used to calculate the comprehensive scores of the measured and predicted data, and the top five optimal PCMs are shown in Table 2, as shown in Figure 6. The highest-scoring three combinations are analyzed and verified using the DSC method, as shown in Figure 7a. The crystallization wind temperature is 0.7589 mw/mg. The initial extrapolated temperature (phase transition temperature) and the final extrapolated temperature obtained from the tangent of the crystallization peak temperature and the DSC curve are 25.7 °C and 48 °C, respectively. The area enclosed by the onset of crystallization, the completion of crystallization, and the crystallization peak temperature represents a phase change latent heat of 74.1 J/g. The results indicate that the predictive results are consistent with the optimal combination obtained from the Taguchi experiment. This demonstrates that machine learning can be utilized to find the optimal ratios of nonlinear small-sample composite phase change materials, providing reliable support for reducing experimental errors and costs in subsequent work.

## 3. Conclusions

The current paper explores the feasibility and effectiveness of using machine learning techniques to construct orthogonal experimental models for predicting full experimental data and finding optimal results. Taguchi experimental data are used as input factors. By analyzing the predictive performance of three machine learning algorithms, such as SVR, RF, and GBDT. The following conclusions are drawn:

The GBDT is more suitable for the thermal performance optimization prediction of gel phase change composite wall materials, including time cooling (TC), latent heat of phase change (ΔH_m_), supercooling degree (ΔT), and phase change temperature (T_m_), which is more suitable to optimal design of PCMs, rather than Support Vector Regression (SVR) and Random Forest (RF). The optimal result predicted by GBDT is Na_2_HPO_4_·12H_2_O + 5% Na_2_SiO_3_·9H_2_O + 12% KCl+ 0.2% Nano-α-Fe_2_O_3_ + 3% C_35_H_49_O_29_, which was verified by experiments.

Due to the limited amount of data in the current paper, automated hyperparameter tuning methods that rely on large datasets (such as grid search and Bayesian optimization) may misidentify optimal parameters because of significant fluctuations in cross-validation results. Moreover, the extensive search spaces can lead to local optima under small sample conditions due to parameter sensitivity. In contrast, manual hyperparameter tuning allows for leveraging domain expertise and data characteristics to focus on adjusting key parameters, thereby enabling more efficient utilization of data information and ensuring the model’s generalization capability to prevent overfitting.

## 4. Materials and Methods

### 4.1. Na_2_HPO_4_·12H_2_O Composite Phase Change Materials

Na_2_HPO_4_·12H_2_O (DHPD, AR) was used as the PCM, while Na_2_SiO_3_·9H_2_O (SMN, AR) was employed as the nucleating agent, and KCL (AR) was applied as the phase transition temperature modifier. All reagents mentioned above were purchased from Sinopharm Chemical Reagent Co., Shanghai, China. C_35_H_49_O_29_ (XG, USP grade), serving as the thickening agent, was supplied by Shanghai Aladdin Biochemical Technology Co., Ltd., Shanghai, China. The thermal conductivity enhancer was α-Fe_2_O_3_ with an average particle size of 30 nm, obtained from Shanghai Xinzhao Welding Materials Co., Ltd., Shanghai, China. All chemicals were used as received without further purification.

To develop PCMs exhibiting low supercooling, an appropriate phase transition temperature, high thermal conductivity, and no phase separation, this study investigated the influence of various dopants on the performance of multicomponent PCMs. The experimental design was formulated using the Taguchi method, with the proportions of SMN, XG, KCl, and nano α-Fe_2_O_3_ selected as key influencing factors, as detailed in Table 3. This was conducted based on our previous research [38].

### 4.2. Framework

**Figure 8:**
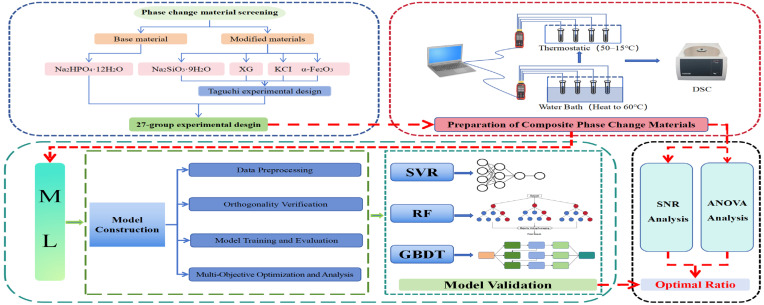
Framework for the preparation of gel PCMs.

To clearly outline the research methodology, Figure 8 presents the proposed prediction framework for the optimal composition of Na_2_HPO_4_·12H_2_O-based gel phase change materials. The workflow mainly consisted of two core stages: (1) the development of 27 composite phase change material groups through Taguchi experimental design, where optimal formulations were identified using signal-to-noise ratio analysis and ANOVA and (2) the fabrication of another 27 composite phase change material groups based on the Taguchi methodology, followed by the construction of three machine learning models to predict the properties of the remaining 54 experimental groups, thereby enabling the identification of the optimal formulation.

### 4.3. Methods

#### 4.3.1. Signal-to-Noise Ratio Analysis and Variance Analysis Method

The SNR method was applied to analyze the experimental data (metrics). SNR functioned as a quantitative indicator for evaluating the performance of composite PCMs and quantifying the influence of selected factors on it. The analysis was carried out based on three types of quality characteristics: higher-the-better (HB), lower-the-better (LB), and nominal-the-best (NB). The corresponding computational formulas are as follows:

(1) $$\eta_{1}=-10\mathrm{lg}\left[\frac{1}{n}\sum_{k=1}^{n}\left(\frac{1}{y_{k}^{2}}\right)\right]$$

(2) $$\eta_{2}=-10\mathrm{lg}\left[\frac{1}{n}\sum_{k=1}^{n}\left(y_{k}^{2}\right)\right]$$

(3) $$\eta_{3}=-10\mathrm{lg}\left[\frac{1}{n}\sum_{k=1}^{n}\left(y_{k}^{2}-m\right)\right]$$

where η is the S/N ratio. n is the number of repetitions of the experimental combination. $y_{i}$ is the experimental response, and m is the mean value of the specific response. In accordance with the “higher-the-better” principle, the latent heat of phase change was calculated; conversely, under the “lower-the-better” principle, the degree of supercooling, thermal conductivity, and phase transition temperature were determined.

Following the calculation of the S/N ratio, analysis of variance (ANOVA) was applied to further analyze the experimental data obtained via the Taguchi method, thereby enabling a quantitative evaluation of the significance of various influencing factors. The primary purpose of ANOVA was to assess experimental errors, identify major sources of variation, and apply statistical techniques to determine the significance levels of control factors. The probability value (*p*-value), calculated using Minitab software 2019, served as a key statistical indicator for assessing the impact of each factor. A factor was considered to have a highly significant (HS) effect when its *p*-value was less than 0.05, whereas a *p*-value greater than 0.05 but less than 0.1 indicates a significant (S) effect.

#### 4.3.2. Candidate Machine Learning Models

(1) Support Vector Regression

Support Vector Regression (SVR) is an extended formulation of Support Vector Machines (SVM) that has been specifically adapted to address regression tasks. The core idea was to construct an optimal hyperplane such that the error of all sample points from this hyperplane did not exceed a specified tolerance (ε). Linear loss was only computed when the deviation surpassed ε, while simultaneously maximizing the “margin” of the hyperplane. The mathematical formulation was as follows:

(4) $$L_{\varepsilon }(y,\;\hat{y})=\left\{\begin{array}{ll} 0, & \mathrm{if}|y-\hat{y}|\leq \varepsilon \\ |y-\hat{y}|-\varepsilon , & \mathrm{otherwise} \end{array}\right.$$

where y is the true value of a sample. $\hat{y}$ is the model’s prediction vector for the sample.

Unlike traditional regression models (such as linear regression), SVR was focused on whether the “deviation between the predicted value and the true value exceeded the threshold ε” rather than directly minimizing all errors. This characteristic made SVR more robust to noise and outliers.

(2) Randon Forest

The fundamental concept of Random Forest was to construct multiple decision trees in parallel by implementing bootstrapping on the available sample data. During the node splitting of each individual tree, a random subset of features was selected to identify the optimal splitting point. The final prediction result was derived from the average of all decision tree outputs in the case of regression or from the majority vote among the trees in the case of classification. The parallel efficiency of Random Forest depended not only on the task scheduling of the computational framework but also on the proper configuration of parameters. For example, controlling parameters, such as n_estimators, max_features max_depth, and min_samples_leaf, was essential. Among these, n_estimators and max_features served as the core for variance control, as follows:

(5) $$V\mathrm{ar}\left(\hat{f}\left(x\right)\right)=\frac{1}{N}V\mathrm{ar}\left(h_{i}\left(x\right)\right)+\left(1-\frac{1}{N}\right)C\mathrm{ov}\left(h_{i}\left(x\right),h_{j}\left(x\right)\right)$$

where N is the total number of decision trees in the random forest. $h_{i}\left(x\right)$ represents the prediction result of the i-th decision tree for sample x. $Var\left(h_{i}\left(x\right)\right)$ indicates the degree of variance in the prediction results of the individual decision tree $h_{i}$; $Cov\left(h_{i}\left(x\right),h_{j}\left(x\right)\right)$ represents the covariance of predictions between two decision trees, $h_{i}$ and $h_{j}$. $\hat{f}\left(x\right)$ represents the model’s prediction vector for the sample set. $Var\left(\hat{f}\left(x\right)\right)$ denotes the prediction variance of the ensemble model.

(3) Gradient Boosting Trees

Gradient Boosting Trees are algorithms based on the Bagging (Bootstrap Aggregating) ensemble learning strategy. Compared to Random Forest (RF), the core idea was to construct multiple decision trees in a sequential manner. Trees were generated sequentially, with each subsequent tree depending on the previous one. The objective was to “fit the residuals (or the negative gradient of the loss function) of the preceding model.” Through iterative adjustments, residuals (the negative gradient of the loss function) were progressively fitted to correct errors. When the iteration met the stopping criteria (residuals are small enough to no longer change), the accumulated “corrections” from all iterations yielded the final model, as expressed by the following formula:

(6) $$F_{M}\left(x\right)=F_{0}\left(x\right)+\sum_{m=1}^{M}\eta \cdot h_{m}\left(x\right)$$

(7) $$F_{0}\left(x\right)=\frac{1}{N}\sum_{i=1}^{N}y_{i}$$

where $F_{M}\left(x\right)$ represents the final prediction result of the gradient boosting tree model for the sample features x after M iterations. $F_{0}\left(x\right)$ denotes the initial prediction model. M indicates the number of iterations. η is the learning rate. $h_{m}\left(x\right)$ represents the weak learner (decision tree) trained during the m-th iteration. m is the counter variable for iteration steps. N is the sample size. $y_{i}$ is the true label of the i-th sample.

#### 4.3.3. Machine Learning Model Construction

The current paper utilized three machine learning algorithms to verify the optimal ratio of composite phase change materials: Support Vector Regression (SVR), Random Forest (RF), and Gradient Boosting Decision Trees (GBDT). Feature engineering could remove irrelevant features to reduce data dimensionality, avoid the “curse of dimensionality”, and lower the computational cost of model training and the risk of overfitting, thus improving prediction accuracy and generalization capability. The current paper created enhanced features to improve model performance, including the addition of interaction terms and quadratic terms. K-fold cross-validation was a data partitioning technique used to evaluate the predictive performance of a model in an unbiased manner. This approach divided the dataset into K mutually exclusive subsets, with one subset serving as the validation set, and the remaining K-1 subsets functioning as the training set in each iteration. The procedure was repeated K times, ensuring that each subset is used exactly once as the validation set. Finally, performance metrics were collected from the K iterations, and their average is calculated as the overall performance measure of the model. In this study, K was set to 5.

Introduce the coefficient of determination (R^2^), mean absolute error (MAE), and root mean square error (RMSE) to evaluate the model’s testing performance, which are calculated as follows:

(8) $$R^{2}=1-\frac{\sum_{i=1}^{n}{\left(y_{i}-{\hat{y}}_{i}\right)}^{2}}{\sum_{i=1}^{n}{\left(y_{i}-\bar{y}\right)}^{2}}$$

(9) $$MAE=\frac{1}{n}\sum_{i=1}^{n}\left|y_{i}-\left.{\hat{y}}_{i}\right|\right.$$

(10) $$RMSE=\sqrt{\frac{1}{n}\sum_{i=1}^{n}{\left(y_{i}-{\hat{y}}_{i}\right)}^{2}}$$

where $y_{i}$ is the true value of the i-th sample. ${\hat{y}}_{i}$ is the predicted value of the i-th sample.

#### 4.3.4. Multi-Objective Optimization and Analysis

The current paper was based on the full factorial combination logic of orthogonal experiments and employed interval optimization methods to obtain its ideal target value. A theoretical full factorial combination of 81 groups was generated, which was compared to the existing measured data of 27 groups. From this, 54 groups that had not been experimentally tested were selected as prediction targets. A trained model was used to predict the combinations that had not been tested, and the predicted data was combined with the measured data to form a complete dataset. Through standardization and the design of a custom scoring function, a comprehensive objective function was established to determine the weights of each factor and identify the optimal combination for comprehensive optimization analysis. Additionally, a heatmap was used to analyze the correlation between factors and target values, providing direction for the quality proportions of modifying materials to be added to the composite materials in subsequent research. The core idea of the interval optimization method was to provide the highest reward when the objective value was within a specific process window (ideal range) and to impose penalties for deviating from that range. According to Equation (11), PCMs were used in the CSG for tomato cultivation, so there was a standard range for their phase change temperature (25–28 °C) to prevent economic losses caused by excessively high or low temperatures for the crops.

(11) $$S_{TM}\left(x\right)=\left\{\begin{array}{ll} 1-0.067\times \left|x-26.5\right| & x\in \left[25,28\right]\\ \max\left(0,0.8-0.1\times \delta \right) & \mathrm{other} \end{array}\right.$$

#### 4.3.5. Model Parameter Settings

In the process of constraining model construction and optimization, parameter adjustment was a core aspect that controlls model complexity and improves fitting performance. Table 4 presents the parameter setting coefficients for the three models in the current paper.

## Figures and Tables

**Table 1:** Experiment L_9_(3^4^) Taguchi hybrid array design and performance indicators.

Group Number	A	B	C	D	TC	H	T	TM
1	3	12	0.1	2	5078	72.95	9.1	26.2
2	3	12	0.1	3	4940	60.51	8.7	27.2
3	3	12	0.1	4	4830	66.81	9.6	26.6
4	3	14	0.2	3	5550	80.88	6.8	34.2
5	3	14	0.2	4	4993	76.04	7.5	27.3
6	3	14	0.2	2	4852	64.35	7.3	25.7
7	3	16	0.3	4	5286	95.73	11.0	38.8
8	3	16	0.3	2	5083	72.13	9.1	31.7
9	3	16	0.3	3	4833	71.07	6.1	25.0
10	4	12	0.3	2	6608	62.68	10.8	20.9
11	4	12	0.3	3	5344	63.67	9.1	24.5
12	4	12	0.3	4	6927	78.73	10.3	34.3
13	4	14	0.1	3	5467	82.19	8.5	26.4
14	4	14	0.1	4	4955	87.42	5.3	34.6
15	4	14	0.1	2	6193	67.78	9.5	25.5
16	4	16	0.2	4	5724	65.29	9.5	25.9
17	4	16	0.2	2	6084	53.36	11.1	29.8
18	4	16	0.2	3	5284	70.53	9.1	32.9
19	5	12	0.2	2	5322	62.97	7.1	22.7
20	5	12	0.2	3	5933	78.46	2.6	25.5
21	5	12	0.2	4	6059	95.52	3.2	25.0
22	5	14	0.3	3	5654	86.14	2.7	24.1
23	5	14	0.3	4	5782	62.74	3.1	28.3
24	5	14	0.3	2	6838	76.46	10.0	27.2
25	5	16	0.1	4	6378	74.15	8.7	35.7
26	5	16	0.1	2	6290	95.52	9.3	38.2
27	5	16	0.1	3	6660	71.26	8.1	32.2

**Table 2:** Five Optimal Combinations.

	A	B	C	D	TC	H	T	TM	Score
1	5%	12%	0.20%	3%	5933	78.5	2.6	25.5	0.795
2	5%	12%	0.20%	4%	6059	95.5	3.2	25.0	0.781
3	5%	12%	0.10%	3%	5931	74.1	2.8	25.7	0.780
4	5%	12%	0.30%	4%	6611	83.7	3.1	29.1	0.776
5	5%	14%	0.30%	3%	5654	86.1	2.7	24.1	0.747

**Table 3:** Factors and amounts of orthogonal tests.

Level	Element			
	A Na_2_SiO_3_·9H_2_O	B KCI	C Nano-α-Fe_2_O_3_	D XG
	(wt%)	(wt%)	(wt%)	(wt%)
1	3	12	0.1	2
2	4	14	0.2	3
3	5	16	0.3	4

**Table 4:** Model Parameter Coefficients.

	GBDT	RF	SVR
1	n_estimators = 100	n_estimators = 100	kernel = rbf
2	max_depth = 3	max_depth = 5	C = 100
3	learning_rate = 0.1	random_state = 42	epsilon = 0.1
4	random_state = 42	n_jobs = −1	gamma = scale

## Data Availability

The original contributions presented in this study are included in this article. Further inquiries can be directed to the corresponding author.
